# Using Next-Generation Sequencing for DNA Barcoding: Capturing Allelic Variation in *ITS2*

**DOI:** 10.1534/g3.116.036145

**Published:** 2016-10-31

**Authors:** Jana Batovska, Noel O. I. Cogan, Stacey E. Lynch, Mark J. Blacket

**Affiliations:** BioSciences Research, Agriculture Victoria, AgriBio Centre for AgriBioscience, Bundoora, Victoria 3083, Australia

**Keywords:** amplicon sequencing, NGS, Culicidae, microsatellites, indels

## Abstract

Internal Transcribed Spacer 2 (*ITS2*) is a popular DNA barcoding marker; however, in some animal species it is hypervariable and therefore difficult to sequence with traditional methods. With next-generation sequencing (NGS) it is possible to sequence all gene variants despite the presence of single nucleotide polymorphisms (SNPs), insertions/deletions (indels), homopolymeric regions, and microsatellites. Our aim was to compare the performance of Sanger sequencing and NGS amplicon sequencing in characterizing *ITS2* in 26 mosquito species represented by 88 samples. The suitability of *ITS2* as a DNA barcoding marker for mosquitoes, and its allelic diversity in individuals and species, was also assessed. Compared to Sanger sequencing, NGS was able to characterize the *ITS2* region to a greater extent, with resolution within and between individuals and species that was previously not possible. A total of 382 unique sequences (alleles) were generated from the 88 mosquito specimens, demonstrating the diversity present that has been overlooked by traditional sequencing methods. Multiple indels and microsatellites were present in the *ITS2* alleles, which were often specific to species or genera, causing variation in sequence length. As a barcoding marker, *ITS2* was able to separate all of the species, apart from members of the *Culex pipiens* complex, providing the same resolution as the commonly used Cytochrome Oxidase I (*COI*). The ability to cost-effectively sequence hypervariable markers makes NGS an invaluable tool with many applications in the DNA barcoding field, and provides insights into the limitations of previous studies and techniques.

In recent years, DNA barcoding has gained popularity as a method to taxonomically identify unknown specimens. DNA barcoding has significant benefits compared to traditional morphological identification, such as differentiating similar-looking species and identifying immature or damaged specimens. The accuracy and efficiency of DNA-based species identification makes it particularly suitable to vector surveillance and biosecurity programs, where specimen identification informs public health predictions and vector control decisions (Armstrong and Ball 2005; Batovska *et al.* 2016).

Mitochondrial Cytochrome Oxidase I (*COI*) is the most common barcoding gene used for animals; however, other ribosomal or nuclear genes are also often used and offer certain advantages. While mitochondrial genes are highly variable and easier to amplify due to their high copy number, nuclear genes exhibit variable rates of substitution that can provide greater resolving power (Lin and Danforth 2004). The Internal Transcribed Spacer 2 (*ITS2*) region of ribosomal DNA has a high copy number and a higher evolutionary rate than most nuclear DNA; however, it can cause alignment problems due to the presence of variable copies (alleles) within individuals (Álvarez and Wendel 2003). When multiple species are being barcoded, it is beneficial to use a ribosomal or other nuclear gene in addition to a mitochondrial gene. These loci are unlinked and evolve separately, thereby giving independent estimates of genetic lineages and relationships. Numerous mosquito barcoding studies have used both mitochondrial and nuclear markers to distinguish species (Foley *et al.* 2007; Paredes-Esquivel *et al.* 2009; Puslednik *et al.* 2012; Bourke *et al.* 2013). *ITS2* is often used in mosquito studies as its high evolutionary rate makes it useful for investigating closely related species, such as those in the *Anopheles* genus (Wilkerson *et al.* 2004; Marrelli *et al.* 2006; Walton *et al.* 2007; Sum *et al.* 2014).

Currently, Sanger sequencing is the most commonly used method to acquire DNA barcodes (Taylor and Harris 2012). While Sanger sequencing can generate reads up to 1000 bases, there are complications that limit its application in DNA barcoding. Sanger sequencing can only process a single DNA template per sample and multiple templates can cause uninterpretable results. This means that DNA regions with size-variable alleles within an individual cannot be sequenced. For instance, the size of *ITS2* varies in many mosquito species, so it must first be cloned and then sequenced in order to overcome this problem (Wesson *et al.* 1992; Severini *et al.* 1996; Aspen *et al.* 2003). Pseudogenes can also create similar issues and have been detected in some mosquito species (Cywinska *et al.* 2006; Wang *et al.* 2012). Sanger sequencing also struggles with long homopolymeric and repetitive regions, often causing difficulties with microsatellites that may be found in barcoding markers, such as *ITS2* (Banerjee *et al.* 2007).

NGS technologies are able to achieve massive parallel sequencing of single DNA molecules, resulting in high-throughput data, unlike Sanger sequencing. Parallel sequencing means that multiple templates are not a challenge, and all regions and variants of the gene are sequenced, allowing the detection of pseudogenes, contaminants, or allelic variation within individual insects (Shokralla *et al.* 2014). Although whole-genome sequencing was one of the initial uses of NGS technology, a variety of methods have since been developed that can generate multilocus sequence data for various applications such as genotyping and phylogenetics. One method, amplicon sequencing, involves tagging a unique barcode onto PCR-amplicons that are then pooled with other tagged amplicons and sequenced (McCormack *et al.* 2013). This multiplexing capability makes NGS a cost-effective method for DNA barcoding.

In this study, we utilize both traditional Sanger sequencing and NGS amplicon sequencing to characterize a region of *ITS2* known to contain microsatellites and indels in mosquitoes. The success of both methods is compared in order to determine their applicability in sequencing hypervariable genes such as *ITS2*. The variability found within *ITS2* is also analyzed and its utility as a DNA barcoding marker for mosquitoes is assessed, compared with previous estimates based on the mitochondrial DNA locus *COI* (Batovska *et al.* 2016).

## Materials and Methods

### Specimen collection and identification

Mosquitoes were collected as part of the Victorian Arbovirus Disease Control Program (VADCP). The methods used to collect, store, and morphologically identify the mosquitoes are described in Batovska *et al.* (2016). In total, 88 mosquito specimens were used in this study, representing 26 species and 12 genera. Specimen information, including trapping locations, can be found in Supplemental Material, Table S1. These specimens were previously used for a DNA barcoding project based upon *COI* (Batovska *et al.* 2016), and the same taxonomic classification has been employed here. Further details of the specimens used for these projects are recorded in the Mosquitoes of Australia – Victoria (MOAV) project on the Barcode of Life Database (BOLD, www.boldsystems.org).

### DNA isolation and ITS2 amplification

A single leg from each mosquito was used for DNA isolation. Magnetic bead-based nucleic acid extraction was performed using the MagMAX DNA Multi-Sample Kit (Life Technologies, Gaithersburg, MD). Sample homogenization and protocol adjustments are described in Batovska *et al.* (2016). DNA extractions were stored at −20°.

A region of *ITS2* (∼350–600 bp) was amplified using the novel primer pair *ITS2*-MOS-F (5′-GCTCGTGGATCGATGAAGAC-3′) and *ITS2*-MOS-R (5′-TGCTTAAATTTAGGGGGTAGTCAC-3′). These new primers are located in the conserved ribosomal DNA regions flanking *ITS2* and were designed from sequences obtained from GenBank (Wesson *et al.* 1992; Toma *et al.* 2000; Linton *et al.* 2001) using Primer3 version 0.4.0 (Untergasser *et al.* 2012). PCR reactions consisted of a total volume of 25 µl: 15.3 µl 1 × bovine serum albumin (BSA), 2.5 µl 10 × ThermoPol Reaction Buffer (New England Biolabs, Beverly, MA), 2 µl 2.5 µM dNTPs, 1.25 µl of each 10 µM/L primer, 0.2 µl 1.0 U *Taq* DNA Polymerase, and 5–15 ng template DNA. The *ITS2* PCR cycle was as follows: 94° for 2 min; 40 cycles of 94° for 30 sec, 51° for 45 sec, and 72° for 45 sec; and then finally 72° for 1 min. The PCR products were verified on a 2% agarose gel.

### Sanger sequencing and data analysis

Size-verified *ITS2* PCR products were enzymatically purified and sequenced commercially in the forward direction on an ABI3730XL by Macrogen (Korea). To determine the overall quality of the sequences, the percentage of bases with a PHRED quality score (Q) above 20 was obtained from the beginning of the sequence to the final adenine peak. For quality trimming, bad quality bases were automatically removed from the 5′ and 3′ ends using a 0.01 error probability limit in Geneious version 8.1 (www.geneious.com, Kearse *et al.* 2012). Any heterozygous bases found in the chromatograms were manually assessed and the base determined using the International Union of Pure Applied Chemistry (IUPAC) codes. Quality-trimmed Sanger sequences were aligned with ClustalW, sequence divergence was calculated (*p*-distance values), and a bootstrap neighbor-joining tree (1000 replicates) was created using MEGA version 6 (Tamura *et al.* 2007).

### NGS and data analysis

The size-verified *ITS2* PCR products were purified using AMPure XP beads (Beckman Coulter, Brea, CA) with a 0.8 × beads ratio in all instances. Universal *Y*-shape adaptors were ligated onto the individual PCR products and excess adaptors were removed using AMPure XP beads. Unique 8 bp barcodes with Illumina P5 and P7 adapters were added onto the ligated products via PCR (× 18 cycles). An AMPure XP bead clean was performed and each sample was then quantified using a NanoDrop 1000 (Thermo Scientific, Waltman, MA). A single equimolar pool was created and then quantified using a Qubit 1.0 fluorometer (Thermo Scientific). Library size was checked with a 2100 Bioanalyzer (Agilent Technologies, Santa Clara, CA). Any remaining adaptor dimers were removed with a further AMPure XP bead clean-up. The pooled sample was sequenced on a MiSeq platform with 2 × 250 bp reads using version 3 chemistry (Illumina, San Diego, CA).

The demultiplexed MiSeq data were trimmed to remove adapters and low quality sequence reads using a custom Perl script. Reads were removed if they had three or more consecutive ambiguous “N” bases; three or more nucleotides (nt) with ≤ Q20; a median Q score of < 20; or a read length of < 150 nt. Overlapping paired reads were merged using PEAR version 0.9.4 (Zhang *et al.* 2013). Contigs (alleles) were assembled with CAP3 version date 12/21/07 (Huang and Madan 1999) using a sequence identity of 95%. The allele with the largest number of reads attributed to it was considered to be the most common allele. All alleles produced were aligned using ClustalW in Geneious. Sequence divergence was calculated (*p*-distance values), and a bootstrap neighbor-joining tree (1000 replicates) was created using MEGA version 6 (Tamura *et al.* 2007). Phobos version 3.3.12 (Mayer 2006) was used to detect microsatellites in the sequences. Microsatellites were defined as 1–6 bp with at least five repeats (Selkoe and Toonen 2006). Using ClustalW, the most common allele for each sample was aligned to the corresponding Sanger sequence in order to verify if the sequence matched. Coverage was also calculated, which was defined as the percentage of the allele covered by good quality Sanger sequence. Sanger and NGS *ITS2* neighbor-joining trees were compared to determine how many individuals were not resolved monophyletically; these included individuals that formed a complex with closely related species or did not resolve monophyletically with conspecifics. A two-by-two table was generated to compare this data, and overall percent agreement was calculated.

### Data availability

All 382 NGS allele sequences are accessible on GenBank (accessions KU495620–KU495708: most common alleles; KX865970–KX866263: other alleles). The most common alleles for each of the specimens are also available on the MOAV project on BOLD (accessions in the range of MOAV001-15–MOAV116-15), as is all associated specimen information. The raw sequencing files for all 88 samples have been deposited at the NCBI SRA database under project ID PRJNA343434.

Individual specimen details are found in Table S1, including trapping locations. Figure S1 compares Sanger and average NGS sequence lengths for all individual specimens. Figure S2 shows the location of microsatellites in all 382 NGS allele sequences. The neighbor-joining analysis of the 88 Sanger sequences can be seen in Figure S3. 

## Results

### Sanger sequencing

**Figure 1:**
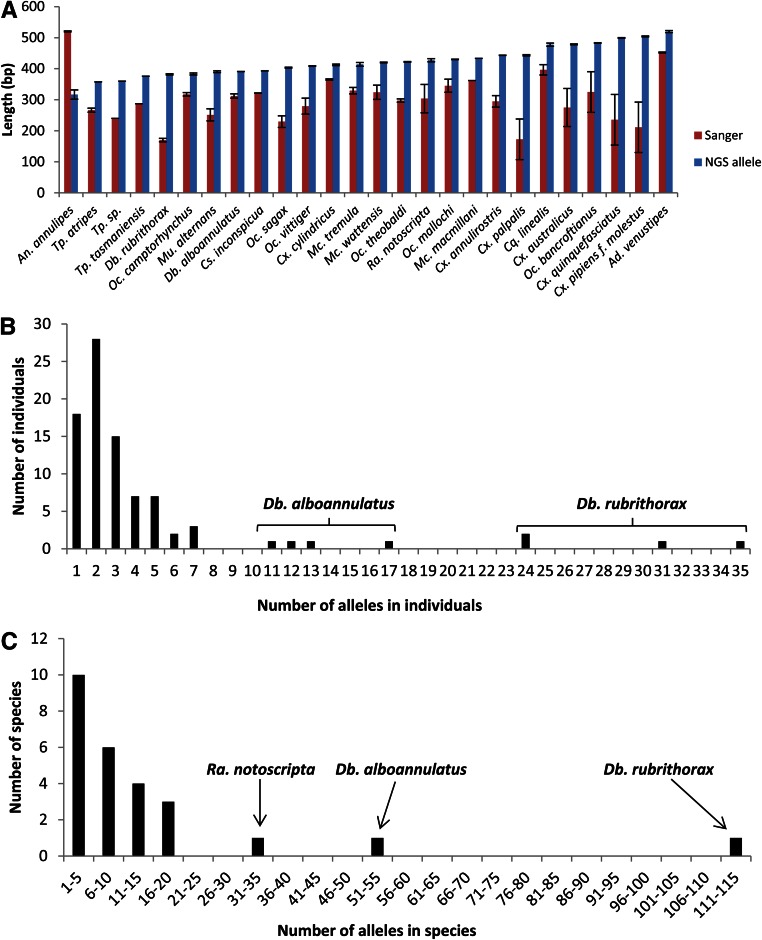
*ITS2* sequence information. (A) Comparison of mean Sanger sequence length post-trimming (red) and mean NGS allele length (blue) between species with standard error shown. (B) Number of alleles per individual shown as a histogram. (C) Number of alleles per species shown as a histogram. NGS, next-generation sequencing.

In total, 88 Sanger sequences were generated for *ITS2*, one for each sample in the forward direction. The quality of the sequences varied among species (Table 1). *Culex cylindricus* and *Aedeomyia venustipes* had consistently good quality sequences, with 91–93 and 92–94% of untrimmed bases > Q20, respectively; whereas other species had poor quality sequences, such as *Dobrotworskyius rubrithorax* with only 59–63% of bases > Q20 (Table 1). Interestingly, some species had both good and bad quality sequences, including *Cx. pipiens* form *molestus* sequences ranging between 33 and 93% of bases > Q20. After quality trimming, sequences were 95–523 bp in length, with the average length and SE for each species shown in Figure 1A, and the length for each individual shown in Figure S1.

**Table 1:** *ITS2* sequence information for individual specimens

		NGS			Sanger		
VAITC	Species Name	No. of Alleles	Size Range (bp)	Average Size (bp)	Bases > Q20 (%)	Post-Trim (bp)	Coverage of NGS (%)
4333	*Aedeomyia venustipes*	1	522	522	94.0	453	86.8
4560A	*Aedeomyia venustipes*	2	509–522	516	93.4	454	88.1
4560B	*Aedeomyia venustipes*	1	522	522	91.8	450	86.2
4309A	*Anopheles annulipes*	2	336–357	347	91.0	518	149.5*^a^*
4309B	*Anopheles annulipes*	2	282–316	299	90.2	520	173.9*^a^*
4309C	*Anopheles annulipes*	1	305	305	91.3	523	171.5*^a^*
4315A	*Coquillettidia linealis*	4	454–485	468	90.1	413	88.3
4315B	*Coquillettidia linealis*	1	482	482	84.8	380	78.8
4332B	*Coquillettidia linealis*	1	483	483	83.2	380	78.7
4342	*Culex annulirostris*	1	442	442	80.9	323	73.1
4344	*Culex annulirostris*	2	444–446	445	70.3	251	56.4
4345	*Culex annulirostris*	3	442–447	444	67.1	251	56.6
4314B	*Culex annulirostris*	2	440–447	444	78.9	328	74.0
4324B	*Culex annulirostris*	4	438–449	442	80.5	277	62.6
4310A	*Culex australicus*	2	476–482	479	84.6	364	76.0
4310B	*Culex australicus*	3	470–478	474	85.3	370	78.0
4326A	*Culex australicus*	1	478	478	36.9	126	26.4
4326B	*Culex australicus*	3	478–486	482	87.4	391	81.1
4326C	*Culex australicus*	2	478–482	480	62.8	124	25.8
4348B	*Culex cylindricus*	5	378–432	410	92.6	366	89.3
4348C	*Culex cylindricus*	4	394–432	408	92.9	370	90.7
4348D	*Culex cylindricus*	7	394–432	418	91.2	361	86.3
4348E	*Culex cylindricus*	2	396–432	414	91.9	364	87.9
4682B	*Culex palpalis*	3	436–446	441	71.3	238	53.9
4683A	*Culex palpalis*	2	442–449	446	55.3	107	24.0
4312A	*Culex pipiens* form *molestus*	2	501–512	507	33.3	99	19.5
4312B	*Culex pipiens* form *molestus*	2	499–508	504	63.7	196	38.9
4339A	*Culex pipiens* form *molestus*	1	506	506	93.0	446	88.1
4339B	*Culex pipiens* form *molestus*	1	501	501	34.1	104	20.8
4318B	*Culex quinquefasciatus*	3	492–504	497	40.8	95	19.1
4318C	*Culex quinquefasciatus*	1	501	501	67.4	299	59.7
4318D	*Culex quinquefasciatus*	1	500	500	34.9	111	22.2
4318E	*Culex quinquefasciatus*	1	499	499	92.2	437	87.6
4903	*Culiseta inconspicua*	1	393	393	89.5	321	81.7
4904	*Culiseta inconspicua*	1	393	393	89.8	322	81.9
4329B	*Dobrotworskyius alboannulatus*	17	376–399	391	84.3	295	75.5
4331A	*Dobrotworskyius alboannulatus*	11	376–397	390	88.4	319	81.7
4331B	*Dobrotworskyius alboannulatus*	12	376–399	392	89.8	327	83.5
4331C	*Dobrotworskyius alboannulatus*	13	376–399	391	81.2	307	78.5
4681	*Dobrotworskyius rubrithorax*	24	318–404	379	62.6	172	45.4
4902	*Dobrotworskyius rubrithorax*	35	318–403	384	60.1	175	45.5
4327A	*Dobrotworskyius rubrithorax*	31	319–401	378	62.3	179	47.3
4327B	*Dobrotworskyius rubrithorax*	24	341–404	386	58.7	154	39.9
4551	*Macleaya macmillani*	2	433–434	434	90.7	362	83.5
4334	*Macleaya tremula*	2	410	410	88.7	337	82.2
4556	*Macleaya tremula*	2	411–433	422	73.4	309	73.2
4905	*Macleaya tremula*	1	410	410	90.1	342	83.4
4335	*Macleaya wattensis*	3	418–420	419	79.9	301	71.8
4558	*Macleaya wattensis*	2	416–426	421	88.3	347	82.4
4323	*Mucidus alternans*	1	392	392	58.9	216	55.1
4328A	*Mucidus alternans*	2	392–396	394	59.4	193	49.0
4328B	*Mucidus alternans*	2	392–396	394	78.2	285	72.3
4336A	*Mucidus alternans*	3	385–394	390	82.9	287	73.5
4336B	*Mucidus alternans*	3	356–396	381	77.3	276	72.4
4308A	*Ochlerotatus bancroftianus*	2	480–482	481	82.0	365	75.9
4308B	*Ochlerotatus bancroftianus*	3	480–491	484	90.0	408	84.4
4308C	*Ochlerotatus bancroftianus*	4	475–491	483	47.6	130	26.9
4308D	*Ochlerotatus bancroftianus*	4	478–491	485	88.1	397	81.9
4330	*Ochlerotatus camptorhynchus*	2	383–387	385	88.4	327	84.9
4341	*Ochlerotatus camptorhynchus*	3	372–387	378	89.9	324	85.8
4347	*Ochlerotatus camptorhynchus*	3	368–387	378	90.1	317	83.8
4304B	*Ochlerotatus camptorhynchus*	1	391	391	83.7	302	77.2
4303	*Ochlerotatus mallochi*	5	418–437	428	89.0	370	86.4
4325	*Ochlerotatus mallochi*	2	425–436	431	73.6	262	60.9
4553	*Ochlerotatus mallochi*	2	425–436	431	89.2	359	83.4
4554	*Ochlerotatus mallochi*	5	425–436	430	89.9	378	87.8
4555	*Ochlerotatus mallochi*	5	425–436	430	86.0	356	82.7
4305A	*Ochlerotatus sagax*	3	401–407	403	65.4	210	52.1
4305B	*Ochlerotatus sagax*	4	400–411	406	65.2	213	52.5
4305C	*Ochlerotatus sagax*	5	388–407	400	78.0	286	71.6
4305D	*Ochlerotatus sagax*	5	400–410	405	66.2	209	51.6
4302A	*Ochlerotatus theobaldi*	2	423–425	424	79.1	286	67.5
4302B	*Ochlerotatus theobaldi*	3	411–428	419	78.4	310	73.9
4302C	*Ochlerotatus theobaldi*	3	419–428	423	80.9	292	69.0
4302D	*Ochlerotatus theobaldi*	2	419–426	423	81.9	302	71.5
4301A	*Ochlerotatus vittiger*	2	406–410	408	89.7	337	82.6
4301B	*Ochlerotatus vittiger*	2	406–408	407	80.8	286	70.3
4301C	*Ochlerotatus vittiger*	4	406–414	410	80.1	282	68.9
4301D	*Ochlerotatus vittiger*	2	406–414	410	70.0	212	51.7
4319A	*Rampamyia notoscripta*	5	426–466	440	87.4	354	80.4
4319B	*Rampamyia notoscripta*	7	365–464	427	50.7	123	28.8
4319C	*Rampamyia notoscripta*	7	351–464	423	81.3	373	88.2
4337A	*Rampamyia notoscripta*	6	339–434	412	82.3	332	80.6
4337B	*Rampamyia notoscripta*	6	412–464	435	84.4	336	77.3
4313	*Tripteroides atripes*	2	356–359	358	86.8	273	76.4
4320	*Tripteroides atripes*	2	356–359	358	84.4	260	72.7
4316A	*Tripteroides sp.*	3	354–364	360	80.8	240	66.7
4349	*Tripteroides tasmaniensis*	1	376	376	83.7	287	76.3

NGS, next-generation sequencing; VAITC, Victorian Agricultural Insect Tissue Collection; No., number.

a See text regarding NGS limitation of resolving full length *An. annulipes* alleles.

### NGS

After bioinformatic analysis, NGS generated 382 unique sequences (alleles) for the 88 samples. When the most common allele was aligned to the corresponding trimmed Sanger sequence, there was a 100% sequence identity match in 85 out of the 88 samples. Of the three remaining samples, the Sanger sequence had a 100% sequence identity match with the second most common allele. The difference in read number between the two most common alleles for these three samples ranged from 1.5 to 27.8% of all reads used for CAP3 analysis.

The alignments also revealed that the Sanger sequences post quality trimming covered a mean of 68.2% (range 19.1–90.7%) of the allele lengths. The mean coverage for each species can be seen in Figure 1A, and the coverage for each individual sample is listed in Table 1 and shown in Figure S1. While coverage varied among species with the aforementioned difference in Sanger sequence quality, generally all alleles were longer than the corresponding Sanger sequence. The only species with a Sanger sequence longer than the allele length was *Anopheles annulipes* (Figure 1A). This occurred because the targeted *ITS2* region in this species is < 500 bp, which meant that the bioinformatic process could not create full-length alleles (the MiSeq read length generated was 2 × 250 bp, so the reads do not overlap the entire fragment and therefore the PEAR program cannot join them). Since the allele lengths are not an accurate representation of the full length of *ITS2* in *An. annulipes*, they were not included in the mean coverage calculation.

It should be noted that *Ad. venustipes* had alleles longer than 500 bp (range 509–522 bp). When the raw reads for these samples were aligned to the corresponding Sanger *ITS2* sequences, reads were identified that began after the primer region. This is indicative of degradation or incomplete extension of the *ITS2* amplicons prior to library preparation, and explains how alleles longer than 500 bp could be formed. However, it appears there is a limit to this process, given that *An. annulipes* has a larger *ITS2* region (601 bp, Bower *et al.* 2009) but does not assemble into a single contig.

**Figure 2:**
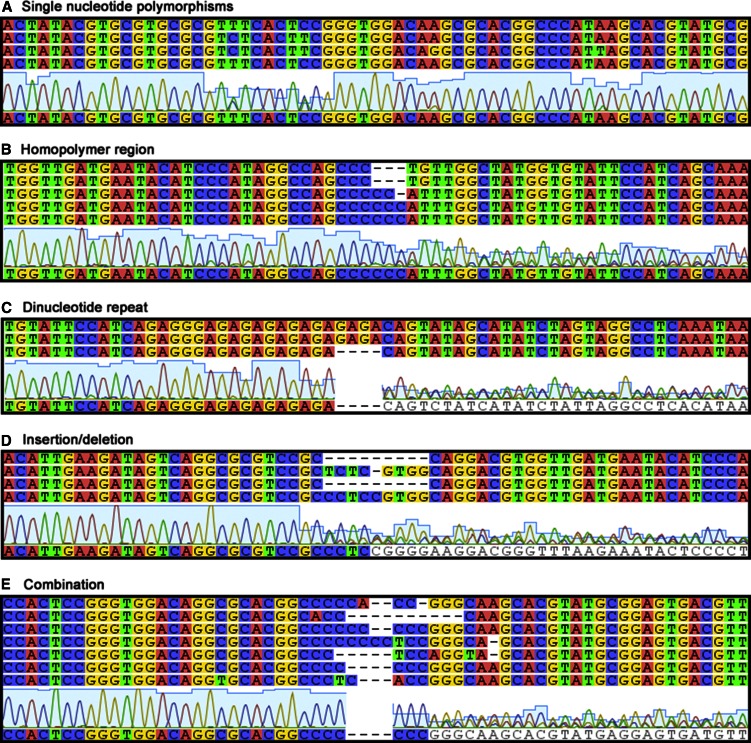
Comparison of sequences generated by Sanger and NGS technology in *ITS2* regions containing different types of polymorphisms observed in this study. (A) Single nucleotide polymorphisms, (B) homopolymer region, (C) dinucleotide repeat, (D) insertion/deletion, and (E) combination. The multiple upper sequences show alleles derived from NGS data, while the lower chromatogram and single accompanying sequence were produced through Sanger sequencing. B and C are examples of microsatellites. NGS, next-generation sequencing.

When examining the Sanger sequences post quality trimming, poor quality sequences were found to have resulted from a variety of allelic differences, including: SNPs, microsatellite regions, and indels. These variable regions were successfully sequenced with NGS and are present in the alleles for each individual. Examples of the effect of different types of polymorphisms in both the Sanger sequences and the alleles are shown in Figure 2.

### Variation within ITS2 alleles

**Figure 3:**
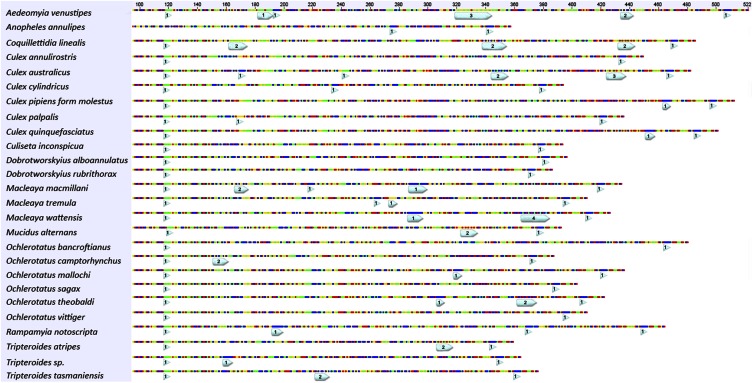
The location of microsatellites within 26 *ITS2* sequences (shown from 100 bp), each representative of a species. The size of the light blue labels are proportionate to repeat length, whereas the number inside the label is representative of the repeat type. Microsatellites within all 382 alleles can be found in Figure S2.

The *ITS2* alleles were found to have many repetitive regions, with microsatellites ranging from 1 to 4 bp in length (Figure 3). In total, the alleles contained 929 homopolymers, 90 dinucleotide, 12 trinucleotide, and four tetranucleotide microsatellites. Many of these microsatellites were specific to species or genera (Figure S2).

The presence of microsatellites and indels led to variation in *ITS2* length within and between individuals (Table 1). The mean allele length for each species is compared in Figure 1A. *Tripteroides atripes* had the shortest *ITS2* alleles (356–359 bp), while *Ad. venustipes* had the longest (509–522 bp).

The number of *ITS2* alleles per individual also varied. Most individuals (*n* = 61, 69%) had one to three alleles, however the two members of the *Dobrotworskyius* genus had substantially more (Table 1). The number of alleles detected in *Db. rubrithorax* ranged from 24 to 35 per individual, resulting in a total of 114 alleles for the species; while the closely related *Db. alboannulatus* also had high allele numbers, with 11–17 per individual and a total of 53 for the species (Figure 1, B and C).

### ITS2 neighbor-joining analysis

**Figure 4:**
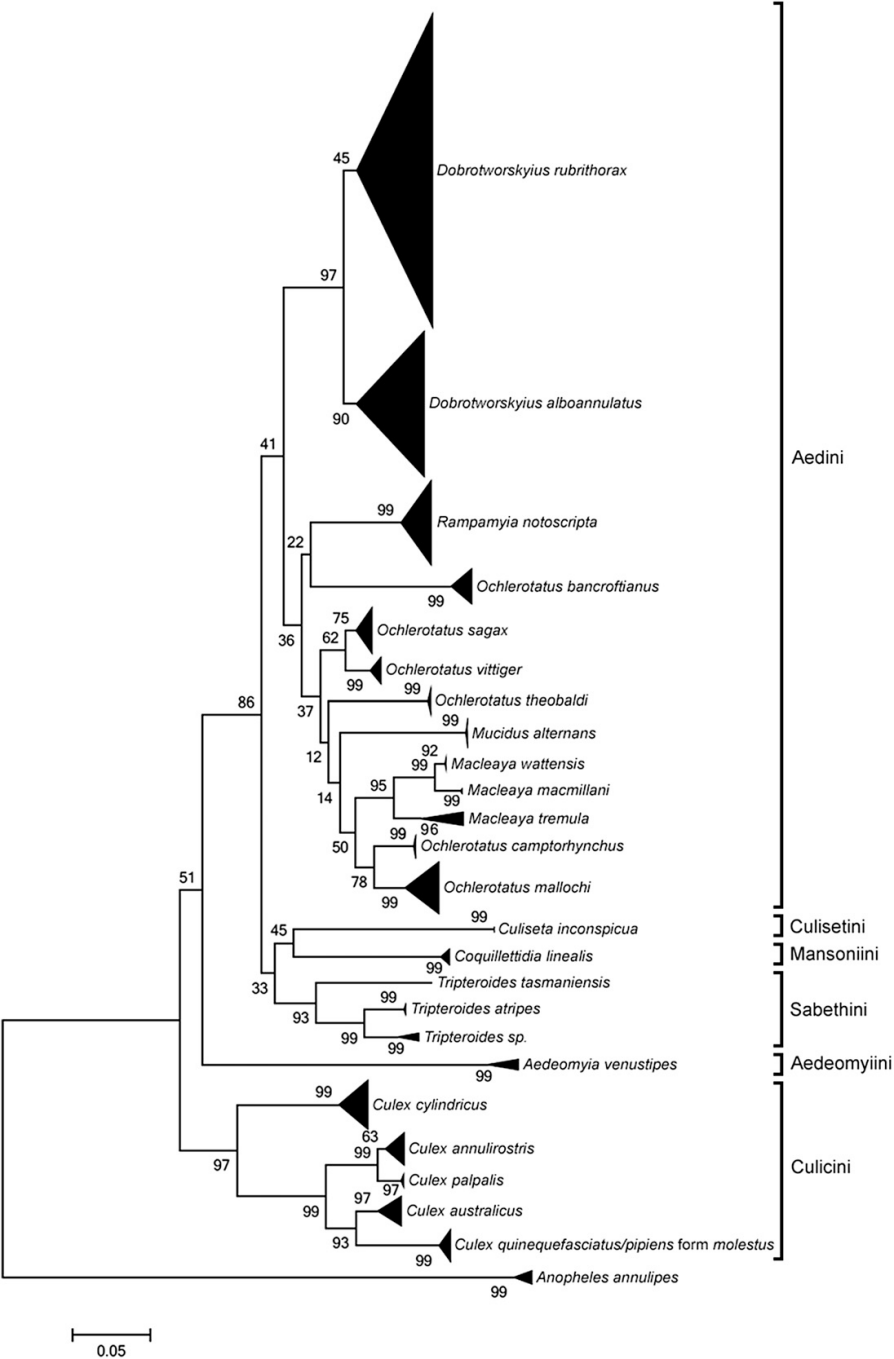
A summarized neighbor-joining tree, with bootstrap support values (%), based on *p*-distance comparisons between 382 *ITS2* contig sequences from 88 mosquito samples. Tribal groups are indicated by brackets.

The neighbor-joining analysis of 88 *ITS2* sequences derived from Sanger sequencing resulted in only 18 of the 26 species resolving monophyletically (Figure S3). All of the *Culex* species failed to form distinct groups (except for *Cx. cylindricus*), as did *Ochlerotatus sagax*, and the *Dobrotworskyius* genus (total of 24 individuals, Table 2). In comparison, NGS sequences provided much greater species resolution, with the neighbor-joining analysis of the 382 alleles resulting in 24 species forming distinct groups on the tree (Figure 4). The only species that failed to form distinct groups were *Cx. pipiens* form *molestus* and *Cx. quinquefasciatus* (total of eight individuals, Table 2). When the number of monophyletically resolved individuals were compared (Table 2), Sanger and NGS had an estimate of agreement of 73%.

**Table 2:** A comparison of the number of individuals that resolved monophyletically using Sanger sequencing and NGS

	NGS		
Sanger	Monophyly	Nonmonophyly	Total
Monophyly	56	0	**56**
Nonmonophyly	24	8	**32**
Total	**80**	**8**	**88**

“Nonmonophyly” included individuals that formed a complex with closely related species or did not resolve monophyletically with conspecifics. These counts provided an estimate of agreement between Sanger and NGS of 73%. NGS, next-generation sequencing.

**Figure 5:**
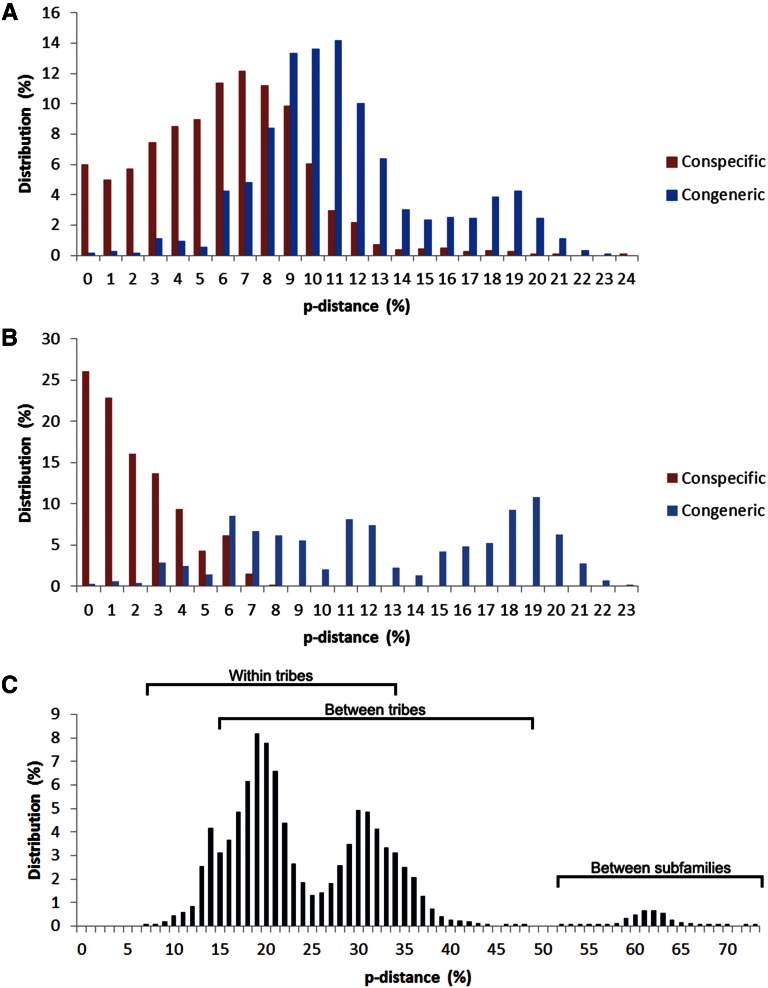
Distribution of percentage difference (*p*-distances) for *ITS2* in different taxonomic categories. Comparison of conspecific and congeneric differences of (A) all 88 samples, and (B) all samples excluding those from the *Dobrotworskyius* genus. (C) Differences between genera in 88 samples, with peaks corresponding to the differences within and between mosquito tribes, and between mosquito subfamilies. Brackets indicate the range of *p*-distances for each group.

Due to the NGS data providing greater species resolution (Figure 4), it was used to calculate the percentage difference (*p*-distance) of different taxonomic categories (Figure 5). The mean conspecific *p*-distance was 6.1% (range 0–24%), compared to 11.4% (range 0–23%) for congeneric divergence (Figure 5A). The mean conspecific *p*-distance decreased substantially from 6.1 to 2% when alleles from the *Dobrotworskyius* genus were removed, revealing a greater differentiation of conspecific and congeneric divergences in other mosquito species (Figure 5B). A DNA barcoding gap was not present (Figure 5B). The mean *p*-distance between genera was 25% (range 7–73%, Figure 5C). Peaks in pairwise comparison values were seen within tribes (mean = 18%), between tribes (mean = 30%), and between subfamilies (mean = 62%, Figure 5C).

## Discussion

### Sanger *vs.* NGS

Our results show that Sanger sequencing is not an appropriate method for characterizing *ITS2* in the majority of mosquito species tested in this study. As shown in Figure 2, there were a variety of polymorphisms in *ITS2* that hampered Sanger sequencing, leading to short sequences post quality trimming that are not suitable for DNA barcoding analyses (Figure S1 and Table 1 and 2). When sequenced with NGS, areas with sequence polymorphisms were fully recovered, revealing the many *ITS2* alleles present in most mosquito species.

Interestingly, some individuals had good quality Sanger sequences, yet had multiple alleles produced from the NGS data (Table 1). This is likely to be due to an abundance of a single dominant allele. While there can be multiple *ITS2* alleles present in an individual, often only one or two are abundant. This was demonstrated by Song *et al.* (2012) when *ITS2* variants from 178 plant species were characterized and it was found that, on average, three of the most prevalent variants accounted for 91% of all *ITS2* copies per species. A similar result was obtained when *Symbiodinium ITS2* variants were sequenced, and the most common copy was on average ∼20 times more prevalent than the second most common copy (Arif *et al.* 2014). Therefore, with the other alleles present in low proportions, Sanger sequencing is able to produce a clean chromatogram. The detection of the rarer alleles in our study demonstrates the sensitivity of the NGS method, as well as generating a more comprehensive sequence data set and resource for these mosquito species.

Conversely, some individuals had disrupted Sanger sequences but only one allele identified in the NGS data (Table 1). One possible cause is the parameters set for CAP3 when assembling the NGS reads, which only produced a contig if sequences were more than 95% similar, meaning that if there was < 5% difference there would still only be one allele. Even this small amount of variation could cause issues with Sanger sequencing considering a single base pair frameshift mutation can disrupt the process. When an individual that had a poor quality Sanger sequence and a single allele was reanalyzed with a 98% cut-off set for CAP3, multiple alleles were produced, indicating that the CAP3 parameters are indeed likely to be responsible for the incongruent data. Other contributors could include the well-documented secondary structure in *ITS2*, quantification issues, and preferential sequencing of primer dimers, which can all result in poor Sanger sequences even if there is only a single *ITS2* allele (Wesson *et al*. 1992; Bertrand *et al*. 2014).

Regardless of the presence of any polymorphic allele issues, the NGS method consistently produced sequences that were closer to the full amplicon length compared to Sanger sequencing (Figure 1A and Figure S1). The only instance in which the Sanger sequence was longer was in the *An. annulipes* species. The *ITS2* region used is larger than 500 bp in this species, and so the 250 bp paired-end reads generated in this study could not overlap to form the full *ITS2* sequence, highlighting a limitation of the NGS method. However, it should be noted that regions marginally larger than the overlapping NGS read length can sometimes be assembled, as seen with *Ad. venustipes*, which had a 509–522 bp *ITS2* region. With other markers, the problem could be solved by ensuring the amplicon being used is no larger than the overlapping NGS read length; but in *ITS2*, suitably conserved PCR priming sites are limited to the flanking ribosomal DNA regions, so reduction of the amplicon size is not feasible. When dealing with size-variable markers, another option is to sequence in only one direction; however, the actual sequence length that is useful for taxonomic assignment would need to be determined (Bertrand *et al.* 2014; Arif *et al.* 2014).

Another advantage of NGS over Sanger sequencing is cost. If Sanger sequencing was used to generate the same level of data as NGS, it would require extensive cloning and sequencing of alleles, which would cost more than a single MiSeq run. Furthermore, Sanger sequencing requires each sample to be processed individually, whereas NGS can sequence all samples simultaneously using internal barcodes attached to Illumina adaptors. The low depth of coverage required means the number of samples can be upscaled to hundreds or thousands, which greatly reduces cost (Adey *et al.* 2010).

### ITS2 variation

The ability to sequence through genetic polymorphisms, such as microsatellites and indels, allowed NGS to characterize *ITS2* allelic diversity in individual mosquitoes. A total of 382 unique *ITS2* alleles were documented, with a mean of 4.3 alleles per individual (range 1–35, Table 1). Concerted evolution is a process that reduces sequence variation among repeats within an individual; however, the presence of intragenomic *ITS2* variation is well-established (Bazzicalupo *et al.* 2013; Bertrand *et al.* 2014; Arif *et al.* 2014). In our study, incomplete concerted evolution appears to be even more prevalent in particular species, with 167 of the 382 alleles attributed to the eight mosquitoes from the *Dobrotworskyius* genus (Figure 1C). Furthermore, although the *Dobrotworskyius* species were recovered in this study, the large differences between alleles within the genus is indicative of incomplete lineage sorting between species (Figure 4 and Figure 5A). Divergent copies of *ITS2* sequences may have been retained over long periods of time and could be useful in elucidating evolutionary relationships (Vargas *et al.* 1999; Wissemann 2002).

In addition to the number of alleles detected, the length of *ITS2* also varied among species, ranging between 356–522 bp. Length was specific to species and caused difficulty in alignment, leading to large differences between some genera and particularly between subfamilies (Figure 5C). Ancient divergences lead to accumulated differences between Anophelinae and Culicinae species, however the degree of distance between these subfamilies using *ITS2* is inflated when compared to mitochondrial genes (Cywinska *et al.* 2006; Versteirt *et al.* 2015; Batovska *et al.* 2016). Alignment-free sequence analysis methods could possibly overcome some of the difficulty associated with comparing alleles of different sizes (Little 2011).

The alignment of alleles revealed that the differences in length were caused by the presence of numerous indels and microsatellites, which were often specific to species (Figure 3 and Figure S2). These polymorphisms are likely to be caused by DNA replication slippage, and their accumulation is furthered by the slow rate of concerted evolution in *ITS2* (Hancock and Vogler 2000). Their fast mutation rate might make them useful for population genetic studies, and species-specific microsatellites could be employed to improve genetic resolution and define boundaries for species determination (Arif *et al.* 2014).

### ITS2 as a barcoding marker for mosquitoes

When using Sanger sequencing, *ITS2* is not a suitable barcoding marker for the species examined in this study, with only 69% of species resolving monophyletically (Figure S3 and Table 2). Due to the polymorphisms found in *ITS2* in these species, Sanger sequencing cannot characterize the region well, apart from when *An. annulipes* species are being sequenced (Figure 1A and Figure S1). However, when using NGS, *ITS2* can separate species with a 96% success rate (Figure 4), despite having overlapping conspecific and congeneric differences, and no clear barcoding gap (Figure 5, A and B). The only species that could not be separated were *Cx. quinquefasciatus* and *Cx. pipiens* form *molestus* (Figure 4). These species are part of the *Cx. pipiens* complex, which is known to be difficult to distinguish using standard barcoding methods (Laurito *et al.* 2013; Pfeiler *et al.* 2013; Batovska *et al.* 2016). In comparison to *COI* as a marker in the same specimens/species (Batovska *et al.* 2016), *ITS2* has similar resolution, with both markers successfully distinguishing all species with the exception of the *Cx. pipiens* complex. Mitochondrial and nuclear genes evolve independently, therefore the concordance between the two datasets observed here helps to confirm the established *COI* species and genera (Lin and Danforth 2004; Reinert *et al.* 2009; Batovska *et al.* 2016). In terms of utility as a DNA barcoding marker for mosquitoes, *COI* appears preferable to *ITS2* as it lacks the alignment issues caused by microsatellites and indels. However, *ITS2* does serve as a useful secondary marker that can now be efficiently obtained using NGS.

### Conclusions

This study found that NGS is the most suitable method for characterizing the *ITS2* region in mosquitoes and this trend is likely to extend into many other insect species and genera. Multiplexing made NGS more efficient and cost-effective than Sanger sequencing, and polymorphic regions were successfully sequenced, revealing the large diversity of *ITS2* alleles present in mosquitoes. The data produced by this study captures the variation in number and size of *ITS2* alleles among species, as well as the abundant microsatellites and indels present. Future studies could use this kind of data to develop markers or investigate evolutionary histories, and lineages with unusual patterns could be examined further, such as the *Dobrotworskyius* genus. As a DNA barcoding marker, *ITS2* functions as well as *COI*; however, *COI* is a superior marker with fewer analytical limitations and complexities. The lack of depth required for NGS amplicon sequencing means that, in the future, a panel of alternative genes could be simultaneously sequenced in a variety of mosquito species in order to find a marker that could successfully separate all species and create well-supported phylogenies. The gene with best resolution may then be used for bulk DNA barcoding, where large pools of mosquitoes could be sequenced and identified, thereby significantly reducing identification time in surveillance programs (Hajibabaei *et al.* 2011). The power and versatility of NGS is revolutionizing many scientific fields, including DNA barcoding, where allelic variation is no longer a limitation but rather an area for discovery.

## Supplementary Material

Supplemental material is available online at www.g3journal.org/lookup/suppl/doi:10.1534/g3.116.036145/-/DC1.
