# Supplementary material for: Using Next-Generation Sequencing for DNA Barcoding: Capturing Allelic Variation in ITS2
Source: G3 (Bethesda). 2016 Oct 31;7(1):19–29. doi: 10.1534/g3.116.036145 (PMC5217108; doi:10.1534/g3.116.036145)

Figure S1 A bivariate plot comparing the expected and Sanger lengths for all 88 mosquito specimens. The correlation coefficient value is -0.1. The expected length is represented by the average NGS length for each individual. The blue symbols are the expected lengths, and the red symbols are the Sanger lengths. The Sanger sequences longer than the expected length represent the *Anopheles annulipes* samples.

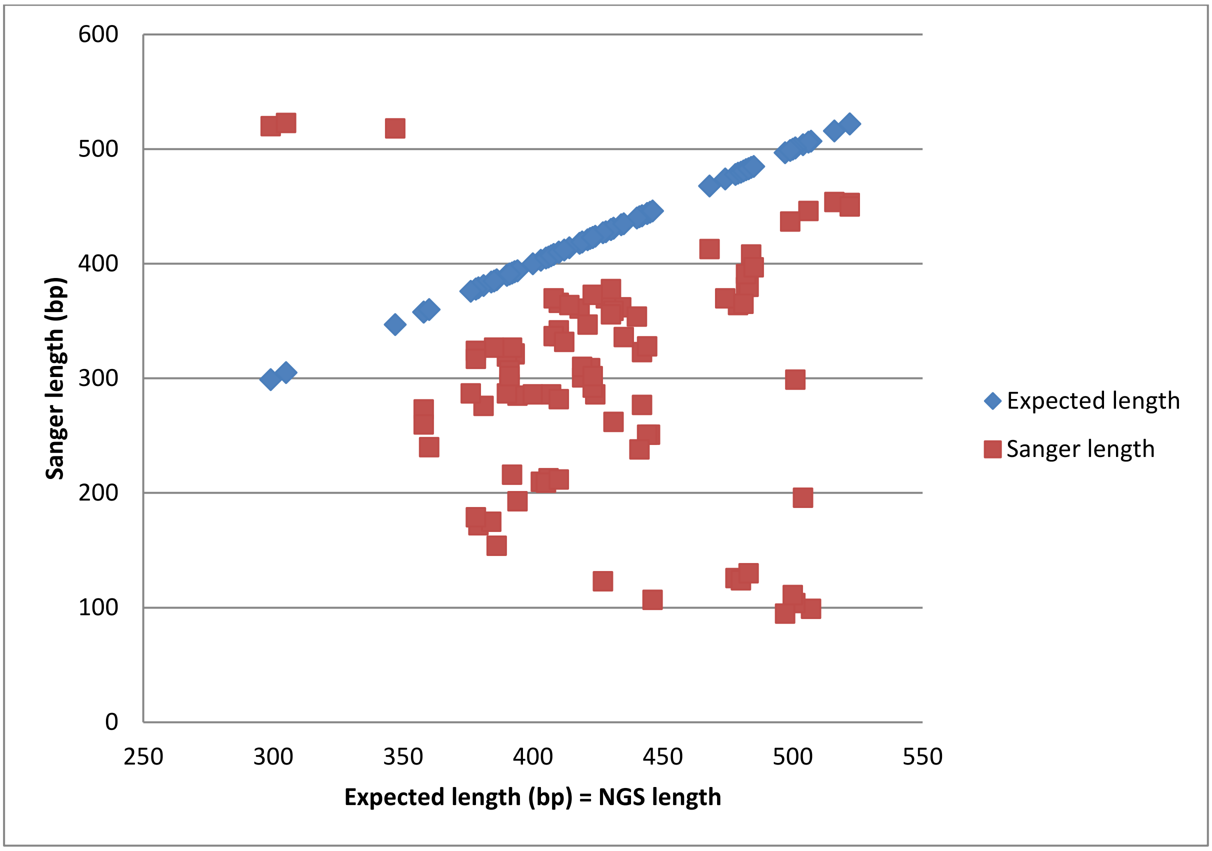

Supplement: Supplementary file 1 [file 19FigureS1.pdf]
