# Supplementary figures and images for: Using Next-Generation Sequencing for DNA Barcoding: Capturing Allelic Variation in ITS2
Source: G3 (Bethesda). 2016 Oct 31;7(1):19–29. doi: 10.1534/g3.116.036145 (PMC5217108; doi:10.1534/g3.116.036145)

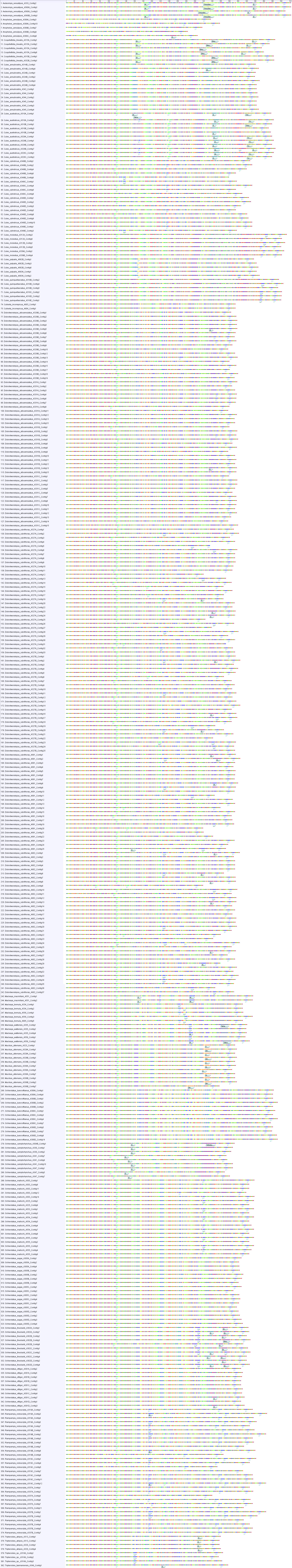

Supplement: Supplementary file 2 [file 19FigureS2.jpg]
