# Supplementary material for: Using Next-Generation Sequencing for DNA Barcoding: Capturing Allelic Variation in ITS2
Source: G3 (Bethesda). 2016 Oct 31;7(1):19–29. doi: 10.1534/g3.116.036145 (PMC5217108; doi:10.1534/g3.116.036145)

Figure S3 A summarised neighbour-joining tree, with bootstrap support values (%), based on p-distance comparisons between ITS2 Sanger sequences from 88 mosquito samples. VAITC numbers are listed next to individuals that were not resolved monophyletically with conspecifics.

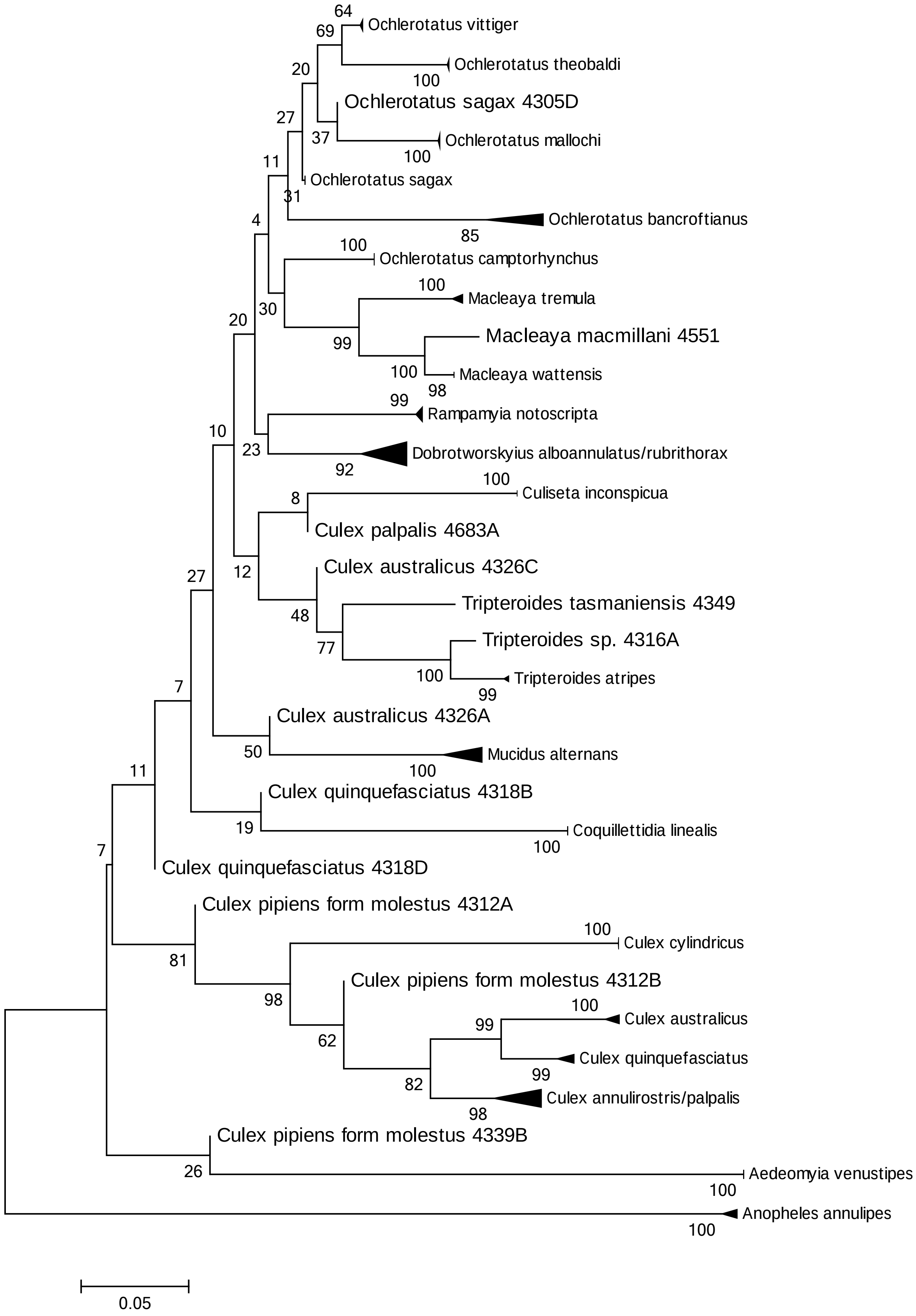

Supplement: Supplementary file 3 [file 19FigureS3.pdf]
