# Supplementary material for: Using Next-Generation Sequencing for DNA Barcoding: Capturing Allelic Variation in ITS2
Source: G3 (Bethesda). 2016 Oct 31;7(1):19–29. doi: 10.1534/g3.116.036145 (PMC5217108; doi:10.1534/g3.116.036145)
Supplement: Supplementary file 4 [file 19TableS1.pdf]

Table S1 Details of specimens used in the study.

ID	n =	Species Name	Traditional Name	City/Town	Latitude	Longitude	Accession Number
4333	1	*Aedeomyia venustipes*	*Aedeomyia venustipes*	Paynesville	37 53 59.83 S	147 43 08 E	KU495621
4560	2	*Aedeomyia venustipes*	*Aedeomyia venustipes*	Meerlieu	37 59 58.64 S	147 22 21 E	KU495620, KU495622
4309	3	*Anopheles annulipes*	*Anopheles annulipes*	Wodonga	36 06 22.60 S	146 52 26 E	KU495623, KU495625-26
4315	2	*Coquillettidia linealis*	*Coquillettidia linealis*	Paynesville	37 53 59.83 S	147 43 08 E	KU495629-30
4332	1	*Coquillettidia linealis*	*Coquillettidia linealis*	Paynesville	37 53 59.83 S	147 43 08 E	KU495628
4314	1	*Culex annulirostris*	*Culex annulirostris*	Wodonga	36 05 21.24 S	146 49 07 E	KU495631
4324	1	*Culex annulirostris*	*Culex annulirostris*	Swan Hill	35 18 34.17 S	143 34 01 E	KU495634
4342	1	*Culex annulirostris*	*Culex annulirostris*	Cobram	35 54 16.12 S	145 38 30 E	KU495635
4344	1	*Culex annulirostris*	*Culex annulirostris*	Toolamba	36 30 43.6 S	145 18 30 E	KU495632
4345	1	*Culex annulirostris*	*Culex annulirostris*	Kerang	35 43 00.71 S	143 54 38 E	KU495633
4310	2	*Culex australicus*	*Culex australicus*	Toolamba	36 30 43.6 S	145 18 30 E	KU495639-40
4326	3	*Culex australicus*	*Culex australicus*	Meerlieu	38 01 15.49 S	147 16 31 E	KU495636-38
4348	4	*Culex cylindricus*	*Culex cylindricus*	Wodonga	36 05 21.24 S	146 49 07 E	KU495645-48
4682	1	*Culex palpalis*	*Culex palpalis*	Paynesville	37 53 59.83 S	147 43 08 E	KU495650
4683	1	*Culex palpalis*	*Culex palpalis*	Kerang	35 42 25.66 S	143 54 24 E	KU495649
4312	2	*Culex pipiens* form *molestus*	*Culex pipiens* form *molestus*	Clydebank	38 04 38.7 S	147 14 40 E	KU495641-42
4339	2	*Culex pipiens* form *molestus*	*Culex pipiens* form *molestus*	Kerang	35 44 25.16 S	143 56 28 E	KU495643-44
4318	4	*Culex quinquefasciatus*	*Culex quinquefasciatus*	Cobram	35 54 16.12 S	145 38 30 E	KU495651-54
4903[a]	1	*Culiseta inconspicua*	*Culiseta inconspicua*	Meerlieu	38 01 15.49 S	147 16 31 E	KU495656
4904[a]	1	*Culiseta inconspicua*	*Culiseta inconspicua*	Meerlieu	38 01 15.49 S	147 16 31 E	KU495655
4329	1	*Dobrotworskyius alboannulatus*	*Aedes alboannulatus*	Armstrong Creek	38 14 09.86 S	144 22 14 E	KU495659
4331	3	*Dobrotworskyius alboannulatus*	*Aedes alboannulatus*	Ocean Grove	38 15 00.77 S	144 31 32 E	KU495657-58, KU495660
4327	2	*Dobrotworskyius rubrithorax*	*Aedes rubrithorax*	Toolamba	36 30 43.6 S	145 18 30 E	KU495663-64
4681	1	*Dobrotworskyius rubrithorax*	*Aedes rubrithorax*	Armstrong Creek	38 14 09.86 S	144 22 14 E	KU495661
4902[a]	1	*Dobrotworskyius rubrithorax*	*Aedes rubrithorax*	Toolamba	36 30 43.6 S	145 18 30 E	KU495662
4551	1	*Macleaya macmillani*	*Aedes macmillani*	Meerlieu	38 03 39.42 S	147 25 39 E	KU495665
4334	1	*Macleaya tremula*	*Aedes tremulus*	Mildura	34 09 42.56 S	142 09 30 E	KU495667
4556	1	*Macleaya tremula*	*Aedes tremulus*	Mildura	34 10 43.34 S	142 08 12 E	KU495666
4905[a]	1	*Macleaya tremula*	*Aedes tremulus*	Mildura	34 09 42.56 S	142 09 30 E	KU495668
4335	1	*Macleaya wattensis*	*Aedes wattensis*	Swan Hill	35 18 34.17 S	143 34 01 E	KU495670
4558	1	*Macleaya wattensis*	*Aedes wattensis*	Cobram	35 52 30.9 S	145 34 09 E	KU495669
4323	1	*Mucidus alternans*	*Aedes alternans*	Toolamba	36 30 43.6 S	145 18 30 E	KU495675

4328	2	*Mucidus alternans*	*Aedes alternans*	Swan Hill	35 18 34.17 S	143 34 01 E	KU495671, KU495674
4336	2	*Mucidus alternans*	*Aedes alternans*	Swan Hill	35 18 34.17 S	143 34 01 E	KU495672-73
4308	4	*Ochlerotatus bancroftianus*	*Aedes bancroftianus*	Wodonga	36 06 22.60 S	146 52 26 E	KU495676-79
4304	1	*Ochlerotatus camptorhynchus*	*Aedes camptorhynchus*	Meerlieu	38 01 15.49 S	147 16 31 E	KU495683
4330	1	*Ochlerotatus camptorhynchus*	*Aedes camptorhynchus*	Point Lonsdale	38 17 12.4 S	144 36 26 E	KU495682
4341	1	*Ochlerotatus camptorhynchus*	*Aedes camptorhynchus*	Mildura	34 10 43.34 S	142 08 12 E	KU495681
4347	1	*Ochlerotatus camptorhynchus*	*Aedes camptorhynchus*	Mildura	34 09 42.56 S	142 09 30 E	KU495680
4303	1	*Ochlerotatus mallochi*	*Aedes mallochi*	Mildura	34 14 58.62 S	142 13 06 E	KU495686
4325	1	*Ochlerotatus mallochi*	*Aedes mallochi*	Mildura	34 14 58.62 S	142 13 06 E	KU495687
4553	1	*Ochlerotatus mallochi*	*Aedes mallochi*	Mildura	34 11 59.79 S	142 12 17 E	KU495685
4554	1	*Ochlerotatus mallochi*	*Aedes mallochi*	Mildura	34 11 59.79 S	142 12 17 E	KU495684
4555	1	*Ochlerotatus mallochi*	*Aedes mallochi*	Mildura	34 09 42.56 S	142 09 30 E	KU495688
4305	4	*Ochlerotatus sagax*	*Aedes sagax*	Kerang	35 42 25.66 S	143 54 24 E	KU495689-92
4302	4	*Ochlerotatus theobaldi*	*Aedes theobaldi*	Toolamba	36 30 43.6 S	145 18 30 E	KU495693-96
4301	4	*Ochlerotatus vittiger*	*Aedes vittiger*	Swan Hill	35 18 34.17 S	143 34 01 E	KU495697-700
4319	3	*Rampamyia notoscripta*	*Aedes notoscripta*	Cobram	35 52 30.9 S	145 34 09 E	KU495701, KU495704-05
4337	2	*Rampamyia notoscripta*	*Aedes notoscripta*	Paynesville	37 53 59.83 S	147 43 08 E	KU495702-03
4313	1	*Tripteroides atripes*	*Tripteroides atripes*	Mildura	34 09 42.56 S	142 09 30 E	KU495708
4320	1	*Tripteroides atripes*	*Tripteroides atripes*	Mildura	34 14 58.62 S	142 13 06 E	KU495707
4316	1	*Tripteroides* sp.	*Tripteroides* sp.	Paynesville	37 53 59.83 S	147 43 08 E	KU495706
4349	1	*Tripteroides tasmaniensis*	*Tripteroides tasmaniensis*	Paynesville	37 53 59.83 S	147 43 08 E	KU495709

[a] = dry-pinned specimens stored in the VAIC.
